# Characteristics of Accelerometry Respondents to a Mail-Based Surveillance Study

**DOI:** 10.2188/jea.JE20100062

**Published:** 2010-11-05

**Authors:** Shigeru Inoue, Yumiko Ohya, Yuko Odagiri, Tomoko Takamiya, Masamitsu Kamada, Shinpei Okada, Catrine Tudor-Locke, Teruichi Shimomitsu

**Affiliations:** 1 Department of Preventive Medicine and Public Health, Tokyo Medical University, Tokyo, Japan; 2 Physical Education and Medicine Research Center Unnan, Unnan, Shimane, Japan; 3 Physical Education and Medicine Research Foundation, Tomi, Nagano, Japan; 4 Walking Behavior Laboratory, Pennington Biomedical Research Center, Baton Rouge, Louisiana, USA

**Keywords:** physical activity, assessment, accelerometer, selection bias

## Abstract

**Background:**

Differences in the characteristics of respondents and nonrespondents to a survey can be a cause of selection bias. The aim of this study was to determine the sociodemographic and lifestyle characteristics of respondents to a field-based accelerometry survey.

**Methods:**

A cross-sectional mail survey was sent to 4000 adults (50% male; age 20 to 69 years) who were randomly selected from the registries of residential addresses of 4 cities in Japan. There were 1508 respondents (responding subsample) to the initial questionnaire. A total of 786 participants from the responding subsample also agreed to wear an accelerometer for 7 days (accelerometer subsample). Age, sex, and city of residence were compared between the accelerometer subsample and all 3214 nonrespondents, including those who did not respond to the initial questionnaire. In addition, multiple logistic regression analyses were used to compare the sociodemographic and lifestyle characteristics of the accelerometer subsample and the 722 respondents who participated in the questionnaire survey but not the accelerometry (questionnaire-only subsample).

**Results:**

As compared with all nonrespondents, the accelerometer subsample included significantly more women, middle-aged and older adults, and residents of specific cities. Multiple logistic regression analyses comparing the accelerometer and questionnaire-only subsamples revealed that participation in the accelerometry survey was greater among nonsmokers (odds ratio, 1.35; 95% confidence interval, 1.02–1.79) and persons who reported a habit of leisure walking (1.56, 1.21–2.01).

**Conclusions:**

Sex, age, city of residence, smoking status, and leisure walking were associated with participation in accelerometry. This response pattern reveals potential selection bias in mail-based accelerometry studies.

## INTRODUCTION

Motion sensors such as accelerometers and pedometers are body-worn assessment devices that objectively capture human movement and are used to assess physical activity behaviors in research and practice. The Japanese Health and Nutrition Survey (JHNS) annually monitors step counts in a representative sample of Japanese.^1^ In the United States, the National Health and Nutrition Examination Survey (NHANES) uses accelerometers to collect step data and also time spent at various activity intensities.^2^^,^^3^ Motion sensors are also being used to motivate increases in physical activity.^4^^,^^5^ Specifically, a systematic review conducted by Bravata et al^4^ suggested that pedometer-based interventions were associated with significant increases in physical activity and decreases in body mass index (BMI) and blood pressure.

The validity of these motion sensors has been evaluated in previous studies. Studies have used oxygen consumption^6^^,^^7^ and the doubly labeled water method^8^ to assess the validity of accelerometers. Pedometer validity has also been evaluated using treadmill walking and track walking.^9^^,^^10^ These studies support the appropriateness of using motion sensors as physical activity assessment tools in research and practice.

Nevertheless, successful practical application of motion sensor technology in field-based survey research requires thorough methodological deliberation. As Trost et al^11^ have indicated, the investigative team must devise a sound plan for collecting and managing the data, including encouragement to join the survey, distribution and collection of devices, decisions on how and when to wear the devices, instructions to participants, processing the data, and decision rules for determining validity of data. One important consideration is selection bias, which is attributable to nonresponse and collection of data deemed invalid because of low compliance to the assigned monitoring protocol. Understanding the sociodemographic characteristics of respondents to motion sensor studies is helpful in establishing sampling and data collection strategies that can then lead to less-biased studies. Further, such information is also helpful in interpreting results of physical activity surveillance studies using pedometers and accelerometers, such as JHNS and NHANES. Unfortunately, there are few data to inform researchers about selection bias in field-based motion sensor studies, including surveillance research.

The purpose of this study was to examine the sociodemographic and lifestyle characteristics of respondents to a mail-based accelerometer survey conducted using a randomly selected sample from 4 cities in Japan. We hypothesized that respondents to the accelerometer survey would report a healthier and more physically active lifestyle than nonrespondents.

## METHODS

### Participants and data collection

This cross-sectional study was conducted from February 2007 through January 2008 and was part of a larger project to investigate the association between neighborhood environment and physical activity.^12^ A total of 4000 adults, age 20 to 69 years, living in 4 cities in Japan (Tsukuba, Koganei, Shizuoka, and Kagoshima) were randomly selected from a registry of residential addresses of each city and stratified by sex, age (20–29, 30–39, 40–49, 50–59, and 60–69 years), and city of residence. The final sample included 2000 subjects of each sex, 800 subjects from each age category, and 1000 subjects from each city. Thus, we obtained the addresses of 100 subjects for every combination of sex, age category, and city.

The surveillance study was divided into 2 parts and was conducted by mail. Letters introducing and describing the study were sent to all 4000 subjects 2 weeks before delivery of the initial survey. The initial survey comprised a questionnaire and an invitation to participate in an additional study that included a 7-day accelerometer survey. Participants signed an informed consent document before answering the questionnaire. This study received prior approval from the Tokyo Medical University Ethics Committee. If respondents to the initial survey consented to join the second survey, then an accelerometer and an additional, related questionnaire were mailed. A call center was set up to manage any survey enquiries during the entire process. Requests to join the survey were mailed out twice in the case of nonresponse. If the survey was returned incomplete, we re-mailed it and asked the respondent to complete it again. A 500-yen book coupon was offered as an incentive to questionnaire respondents, and an additional 500-yen book coupon was offered to accelerometer respondents.

As a result of these strategies, 1508 (37.7%) adults responded to the initial survey (responding subsample) of 4000 residents originally approached to participate (total sample). The offer to join the subsequent accelerometer survey was accepted by 886 individuals from the responding subsample. However, this subsample of 886 included participants who responded to only the questionnaire portion of this second survey and those who wore the accelerometers improperly, eg, did not wear the accelerometer for at least 4 days (details below). After data cleaning, valid accelerometer data (details below) were obtained from 786 participants (accelerometer subsample; final valid respondent rate, 19.7%). Ultimately, there were 3214 nonrespondents (total sample minus accelerometer subsample) to accelerometry, including 722 questionnaire-only participants (questionnaire-only subsample). The participant flow is shown in the Figure.

**Figure.**
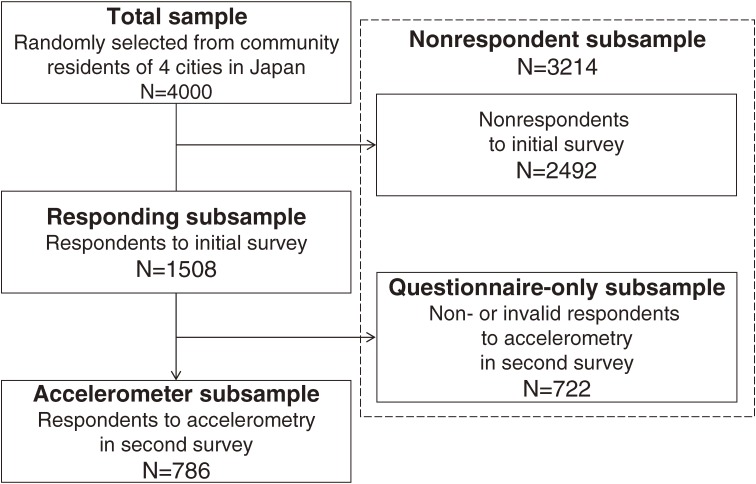
Flow of participants

### Accelerometer survey and data procedure

Participants were asked to wear an accelerometer (Lifecoder EX, 4-second version, Suzken Company, Nagoya, Japan), which included a step-counting function, for 7 consecutive days on their waist throughout the day, except when sleeping or in water (eg, when taking a bath or swimming). This motion sensor has been validated relative to total energy expenditure^8^ and step count.^13^ A record was considered valid when the participant wore the device at least 10 hours a day.^2^ Non-wear was defined as the continuous absence of an acceleration signal for 30 minutes or longer. As in previous studies,^2^^,^^11^ the accelerometer subsample was ultimately defined as those respondents who provided 4 or more valid days of accelerometer data.

### Sociodemographic and lifestyle variables

Sex, age, and city of residence for every subject were obtained from the registry of residential addresses. Information regarding years of education, employment status, marital status, self-rated health, smoking, alcohol intake, walking behavior, body weight, and height was obtained by self-report in the initial survey. BMI (kg/m^2^) was calculated from self-reported body weight and height. Self-rated health was measured with a single item that asked participants to rate their health. Using a 5-point scale (excellent, very good, good, fair, poor), participants were asked to choose the most suitable response for the statement, “In general, you would say that your health is _______.” For smoking and alcohol intake, the questions from the JHNS 2007^1^ were used. The JHNS 2007 asked participants about the frequency of alcohol consumption per week and the amount consumed per day. In the present study, we asked for information on frequency only. Regarding smoking, participants were asked, “Do you currently have a smoking habit?” The choices were every day, sometimes, and never in the last month. As for walking behavior, participants were asked to report their walking frequency (days/week) and duration (minutes/day) for 6 purposes (commuting to work, commuting to school, walking during work, walking for daily errands, walking for leisure, or walking for other purposes). Total walking time (minutes/week) was calculated as the sum of the product of frequency-multiplied duration for the 6 walking purposes. In this study, we also separately examined participation in walking for leisure (yes or no), because this type of walking is considered the most common form of volitional exercise and may be related to motivation to participate in the accelerometer assessment.

### Statistical analysis

Descriptive statistics were used to characterize the total sample, nonrespondent subsample (nonrespondents to the initial survey plus questionnaire-only subsample), and accelerometer subsample. Sex, age, and city of residence were compared between the nonrespondent subsample and accelerometer subsample using the chi-square test. We also used the chi-square test to compare the accelerometer subsample with the questionnaire-only subsample for all 12 sociodemographic and lifestyle variables, including sex, age, city of residence, education, employment status, BMI, and total walking time.

The accelerometer subsample and questionnaire-only subsample were also compared for sociodemographic and lifestyle differences using multiple logistic regression analyses. The sociodemographic and lifestyle variables (independent variables) were sex, age (20–29, 30–39, 40–49, 50–59, or 60+ years), city of residence, years of education (≤12 years or >12 years), employment status (<40 hours/week or ≥40 hours/ week), marital status (married or not married), BMI (<25 kg/m^2^ or ≥25 kg/m^2^), self-rated health (excellent, very good, or good vs fair or poor), smoking (current smoker or not current smoker), alcohol intake (regular drinker, ie, drinking once a week or more, or not a regular drinker), walking for leisure (yes or no), and total walking time (active walker, ie, walking ≥150 min/week, or not an active walker).^14^ Odds ratios for a valid accelerometry response with respect to sociodemographic and lifestyle variables were calculated and adjusted for all other variables.

For all analyses, a *P* value of less than 0.05 was considered to indicate statistical significance. All statistical analyses were performed with SPSS 17.0J for Windows (SPSS Inc., Tokyo, Japan).

## RESULTS

Table 1 shows the characteristics of the total sample, nonrespondent subsample (nonrespondents to the initial survey plus the questionnaire-only subsample), and accelerometer subsample. The accelerometer subsample included 46.4% men; mean age ± standard deviation (SD) was 48.5 ± 13.6 years. The mean step count for this sample was 8476 steps/day, which was higher than the mean of 6839 steps/day noted in the Japanese Health and Nutrition Survey,^1^ although the age distributions of the studies differed. Comparison of the nonrespondent subsample and accelerometer subsample was possible only for sex, age, and city of residence. The accelerometer subsample included significantly more women and more middle-aged and older adults. City of residence also differed between the nonrespondent subsample and accelerometer subsamples. Regarding the comparison of the accelerometer subsample and questionnaire-only subsample, 5 variables (age, smoking, alcohol intake, walking for leisure, and total walking time for any purposes) were significantly different. As compared with questionnaire-only respondents, those in the accelerometer subsample were more likely to be middle-aged, nonsmoking, and regular drinkers, and to report a leisure walking habit and longer total walking time (≥150 min/week).

**Table 1.** Characteristics of the total sample, nonrespondents to the initial survey, questionnaire-only subsample, nonrespondent subsample, and accelerometer subsample

	Total sample		Nonrespondents to initial survey (N_0_)		Questionnaire-only subsample (Q)		Nonrespondent subsample (N = N_0_ + Q)		Accelerometer subsample (A)		*P* value^a^ (N vs A)	*P* value^a^ (Q vs A)
	*n* = 4000		*n* = 2492		*n* = 722		*n* = 3214		*n* = 786			
					
	*n*	%	*n*	%	*n*	%	*n*	%	*n*	%		
Sex												
Men	2000	50.0	1321	53.0	314	43.5	1635	50.9	365	46.4	0.026	0.250
Women	2000	50.0	1171	47.0	408	56.5	1579	49.1	421	53.6		
Age, years												
60–	800	20.0	383	15.4	208	28.8	591	18.4	209	26.6	<0.001	0.005
50–59	800	20.0	465	18.7	163	22.6	628	19.5	172	21.9		
40–49	800	20.0	489	19.6	125	17.3	614	19.1	186	23.7		
30–39	800	20.0	581	23.3	99	13.7	680	21.2	120	15.3		
–29	800	20.0	574	23.0	127	17.6	701	21.8	99	12.6		
City of residence												
Tsukuba	1000	25.0	618	24.8	183	25.3	801	24.9	199	25.3	0.003	0.064
Koganei	1000	25.0	600	24.1	170	23.5	770	24.0	230	29.3		
Shizuoka	1000	25.0	610	24.5	196	27.1	806	25.1	194	24.7		
Kagoshima	1000	25.0	664	26.6	173	24.0	837	26.0	163	20.7		
Education, years												
>12	N/A		N/A		400	56.3	N/A		478	61.0	N/A	0.060
≤12	N/A		N/A		311	43.7	N/A		305	39.0	N/A	
Employment status												
≥40 h/week	N/A		N/A		335	48.8	N/A		385	50.1	N/A	0.622
<40 h/week	N/A		N/A		351	51.2	N/A		383	49.9	N/A	
Marital status												
Married	N/A		N/A		538	75.1	N/A		607	77.5	N/A	0.278
Not married	N/A		N/A		178	24.9	N/A		176	22.5	N/A	
BMI, kg/m^2^												
<25	N/A		N/A		582	81.6	N/A		629	80.2	N/A	0.492
≥25	N/A		N/A		131	18.4	N/A		155	19.8	N/A	
Self-rated health												
Good	N/A		N/A		378	52.8	N/A		419	53.4	N/A	0.801
Poor	N/A		N/A		338	47.2	N/A		365	46.6	N/A	
Smoking												
No	N/A		N/A		487	73.7	N/A		585	79.2	N/A	0.016
Yes	N/A		N/A		174	26.3	N/A		154	20.8	N/A	
Alcohol intake												
No/Not regularly	N/A		N/A		423	59.1	N/A		412	52.8	N/A	0.015
Regularly	N/A		N/A		293	40.9	N/A		368	47.2	N/A	
Walking for leisure^b^												
Yes	N/A		N/A		211	29.8	N/A		312	40.0	N/A	<0.001
No	N/A		N/A		498	70.2	N/A		468	60.0	N/A	
Total walking time^b^, min/week												
≥150	N/A		N/A		422	61.3	N/A		515	68.0	N/A	0.008
<150	N/A		N/A		266	38.7	N/A		242	32.0	N/A	
Step count^c^, steps/day												
Mean ± SD	N/A		N/A		N/A		N/A		8474 ± 3368		N/A	N/A
Total energy expenditure^c^, kcal/day												
Mean ± SD	N/A		N/A		N/A		N/A		1895 ± 309		N/A	N/A

Abbreviations: N/A, not applicable; SD, standard deviation.

Total numbers of participants are not always equal because of missing values.

^a^The chi-square test was used to compare sociodemographic and lifestyle variables between groups (N vs A, Q vs A).

^b^Assessed by questionnaire.

^c^Assessed by accelerometer.

In logistic regression analysis (Table 2), age, smoking, and walking for leisure were significantly associated with accelerometry participation. Odds ratios for the accelerometry subsample were significantly higher among those aged 30 to 39 years (odds ratio, 1.60; 95% confidence interval, 1.04–2.49), those aged 40 to 49 years (1.79, 1.16–2.75), nonsmokers (1.35, 1.02–1.79), and leisure walkers (1.56, 1.21–2.01). That is, individuals with the above characteristics were significantly more likely to participate in the accelerometer portion of the surveillance study. Some sex differences were observed in stratified analyses. As compared with the questionnaire-only subsample, male respondents in the accelerometer subsample were more likely to be nonsmokers and leisure walkers, whereas female respondents were more likely to be older and leisure walkers.

**Table 2.** Odds ratios for response to accelerometry, by sociodemographic and lifestyle variables, as compared with questionnaire-only respondents

	Overall respondents			Men			Women		
	*n* = 1508			*n* = 679			*n* = 829		
			
	OR^a,b^	95% CI	*P* value	OR^a,b^	95% CI	*P* value	OR^a,b^	95% CI	*P* value
Sex									
Men	1.16	(0.88, 1.52)	0.287						
Women	1.00								
Age, years									
60–	1.13	(0.72, 1.76)	0.606	0.78	(0.38, 1.63)	0.509	1.32	(0.73, 2.39)	0.353
50–59	1.17	(0.75, 1.81)	0.484	0.72	(0.36, 1.46)	0.368	1.50	(0.84, 2.68)	0.166
40–49	1.79	(1.16, 2.75)	0.008	1.41	(0.71, 2.80)	0.323	1.89	(1.07, 3.34)	0.028
30–39	1.60	(1.04, 2.49)	0.034	0.83	(0.41, 1.70)	0.613	2.31	(1.32, 4.07)	0.004
–29	1.00			1.00			1.00		
City of residence									
Tsukuba	1.08	(0.78, 1.50)	0.648	1.12	(0.69, 1.83)	0.644	1.04	(0.66, 1.64)	0.864
Koganei	1.27	(0.91, 1.77)	0.154	1.06	(0.64, 1.77)	0.815	1.38	(0.88, 2.16)	0.159
Shizuoka	1.00	(0.72, 1.39)	0.992	0.89	(0.55, 1.45)	0.649	1.08	(0.69, 1.69)	0.748
Kagoshima	1.00			1.00			1.00		
Education, years									
>12	1.01	(0.79, 1.30)	0.918	1.41	(0.97, 2.04)	0.069	0.78	(0.55, 1.10)	0.160
≤12	1.00			1.00			1.00		
Employment status									
≥40 h/week	1.00	(0.77, 1.31)	0.990	1.18	(0.77, 1.83)	0.447	0.86	(0.59, 1.24)	0.411
<40 h/week	1.00			1.00			1.00		
Marital status									
Married	1.00	(0.73, 1.37)	0.991	1.27	(0.75, 2.15)	0.382	0.83	(0.54, 1.29)	0.413
Not married	1.00			1.00			1.00		
BMI, kg/m^2^									
<25	0.84	(0.63, 1.13)	0.256	0.81	(0.55, 1.19)	0.284	0.92	(0.57, 1.48)	0.724
≥25	1.00			1.00			1.00		
Self-rated health									
Good	0.95	(0.75, 1.20)	0.643	0.92	(0.65, 1.29)	0.612	0.99	(0.71, 1.38)	0.943
Poor	1.00			1.00			1.00		
Smoking									
No	1.35	(1.02, 1.79)	0.038	1.46	(1.02, 2.08)	0.038	1.16	(0.71, 1.90)	0.548
Yes	1.00			1.00			1.00		
Drinking									
Regularly	1.22	(0.95, 1.57)	0.115	1.17	(0.82, 1.67)	0.381	1.25	(0.87, 1.79)	0.229
No/Occasionally	1.00			1.00			1.00		
Walking for leisure									
Yes	1.56	(1.21, 2.01)	0.001	1.64	(1.10, 2.44)	0.015	1.48	(1.05, 2.09)	0.025
No	1.00			1.00			1.00		
Total walking time, min/week									
≥150	1.23	(0.96, 1.58)	0.096	1.42	(0.98, 2.07)	0.065	1.12	(0.80, 1.57)	0.515
<150	1.00			1.00			1.00		

^a^OR: odds ratio.

^b^Odds ratios were adjusted for all other variables shown in the table.

## DISCUSSION

The results of this study indicate that response rates to the accelerometer survey were higher among women than men, and among middle-aged and older adults than younger adults. This suggests that selection bias in surveys that use motion sensors systematically results in underrepresentation of men and younger people. The city of residence was also related to the response rate. We speculate that residents who live near the research center (Koganei is in Tokyo and Kagoshima is the farthest from Tokyo) might be more willing to participate in the study. In multivariate analyses comparing the accelerometer and questionnaire-only subsamples, there was a significantly higher response rate to wearing the accelerometer in adults who were middle-aged vs young, nonsmokers vs smokers, and leisure walkers vs non-leisure walkers after adjustment for other sociodemographic and lifestyle variables. These results must be carefully interpreted, since we were not able to compare the characteristics of the accelerometer subsample with those of the original total sample. However, these findings suggest that survey respondents who agreed to wear an accelerometer tended to be nonsmokers and more active walkers. That is, there may be a selection bias leading to overestimation of physical activity levels of populations assessed by an accelerometer or a pedometer in field studies. Although we began our analysis with this hypothesis, there was very little prior evidence to support this assumption.

Previously, Harris et al^15^ conducted a randomized controlled trial of different recruitment strategies to a physical activity study using 560 patients 65 years or older who were registered with a primary care center in the United Kingdom. Participants who responded to the accelerometer assessment portion of that study reported higher physical activity, such as walking, gardening, and heavy housework, and had significantly more health problems, such as chronic pain and chronic diseases, than did nonrespondents. They also reported a faster walking speed and positive attitudes towards activity. Although the setting and age of the target population of this earlier study differ from those of the current analysis, the results were similar: active people were willing to participate in the accelerometer survey. A difference was observed in health status. In our study, health status as measured by self-rated health was not associated with accelerometry response. Because the direction and extent of bias likely differs by target population and study setting, further evidence is needed to understand the impact of selection bias in surveys employing motion sensors, if researchers and practitioners are to effectively implement such strategies and more accurately interpret the results.

Although selection biases are inevitable in field studies, efforts to decrease their impact by increasing the response rate are important. Edwards et al^16^ reviewed studies regarding survey methods to obtain better response rates to postal and electronic questionnaire research. They concluded that effective strategies including monetary incentives, recorded delivery, use of a teaser on the envelope (eg, a comment suggesting to participants that they may benefit if they open it), a more interesting questionnaire topic, pre-notification, follow-up contact, shorter questionnaires, providing a second copy of the questionnaire at follow-up, university sponsorship, handwritten addresses, stamped return envelopes, and assurance of confidentiality. These strategies would also likely be effective in terms of increasing response rates to accelerometer and pedometer surveys. Some of these strategies such as monetary incentives, pre-notification, follow-up contact, university sponsorship, stamped return envelopes, and assurance of confidentiality were adopted in this study. The study might have had a higher response rate and been less biased if we had used additional strategies, such as an envelope teaser, a more interesting invitation, reminder calls, providing clear instructions on how to use the device, providing an example of result output, and designing a better survey schedule that made responses easy for subjects. Although feasible strategies differ by study setting, the results of the present study should encourage researchers to adopt these strategies to increase response rate in field studies using these devices.

There are some limitations in this study. Firstly, we compared most of the independent variables of the accelerometer subsample with the questionnaire-only subsample. For the purpose of this study, it would be ideal to compare the accelerometer and nonrespondent subsamples. However, we only had information on age, sex, and city of residence for the nonrespondent subsample. In this study, the response rate to the initial survey was not high. We must consider the possibility that the questionnaire-only subsample itself was biased from the nonrespondent subsample. Second, this study was a mail survey based upon a random sample of community residents. Our results would, of course, be most generalizable to similar study settings. Results may differ depending on the study setting, purpose, and target population. More evidence of biases in physical activity assessment using objective monitoring devices administered in various study settings is needed to clarify these issues. Thirdly, the characteristics of respondents might be different depending on survey schedule. For example, in this study, we divided the survey into 2 parts. However, the results might have differed if we had requested questionnaire and accelerometry data at the same time.

In spite of these limitations, this study provides important initial evidence suggesting selection bias in accelerometer surveys. The results of this study, which show the characteristics of persons who tend to participate in such surveys, have implications for conducting and interpreting the findings of physical activity studies using accelerometers and pedometers.

In conclusion, women and middle-aged and older adults were more likely to join an accelerometer survey delivered by mail. Participants who responded not only to the questionnaire but also to the accelerometer survey tended to report a healthier and more physically active lifestyle than did questionnaire-only participants. This response pattern reveals potential for selection bias in mail-based accelerometry surveillance studies. It is important to develop strategies to increase overall response rates and address this bias.
